# Elevated *p*CO_2_ enhances bacterioplankton removal of organic carbon

**DOI:** 10.1371/journal.pone.0173145

**Published:** 2017-03-03

**Authors:** Anna K. James, Uta Passow, Mark A. Brzezinski, Rachel J. Parsons, Jennifer N. Trapani, Craig A. Carlson

**Affiliations:** 1 Marine Science Institute, Department of Ecology, Evolution, and Marine Biology, University of California, Santa Barbara, California, United States of America; 2 Bermuda Institute of Ocean Science (BIOS), St. George’s, Bermuda; University of Connecticut, UNITED STATES

## Abstract

Factors that affect the removal of organic carbon by heterotrophic bacterioplankton can impact the rate and magnitude of organic carbon loss in the ocean through the conversion of a portion of consumed organic carbon to CO_2_. Through enhanced rates of consumption, surface bacterioplankton communities can also reduce the amount of dissolved organic carbon (DOC) available for export from the surface ocean. The present study investigated the direct effects of elevated *p*CO_2_ on bacterioplankton removal of several forms of DOC ranging from glucose to complex phytoplankton exudate and lysate, and naturally occurring DOC. Elevated *p*CO_2_ (1000–1500 ppm) enhanced both the rate and magnitude of organic carbon removal by bacterioplankton communities compared to low (pre-industrial and ambient) *p*CO_2_ (250 –~400 ppm). The increased removal was largely due to enhanced respiration, rather than enhanced production of bacterioplankton biomass. The results suggest that elevated *p*CO_2_ can increase DOC consumption and decrease bacterioplankton growth efficiency, ultimately decreasing the amount of DOC available for vertical export and increasing the production of CO_2_ in the surface ocean.

## Introduction

Marine heterotrophic bacterioplankton play a key role in the biogeochemical cycling of carbon through their use of dissolved organic carbon (DOC) [1]. These communities convert a portion of consumed DOC into biomass and respire the remainder to CO_2_, thereby, decreasing total organic carbon concentrations in the ocean through the production of CO_2_. Via air—sea exchange, the production of CO_2_ in the surface ocean could lead to the loss of carbon from the ocean [2]. DOC that escapes bacterioplankton consumption and persists on the timescales of months to years has the potential to be exported out of the surface ocean by physical processes and represents a major export pathway in the biological pump [3, 4, 5]. Thus, factors that affect bacterioplankton consumption of DOC impact surface-ocean carbon inventories and the amount of organic carbon available for export as either DOC or organic carbon associated with bacterioplankton biomass.

How ocean acidification affects marine bacterioplankton directly or indirectly remains a topic of interest [6]. Recent mesocosm and culture experiments comprised of photoautotrophs, heterotrophic bacterioplankton, and grazers have demonstrated clear effects of elevated *p*CO_2_ on bacterioplankton growth [7–9] and extracellular enzyme activities [9–13]. Bacterioplankton protein production [9] and abundance [10] were shown to increase as a function of elevated *p*CO_2_, while Motegi et al. [8] observed no effect of *p*CO_2_ on other aspects of bacterioplankton activity (i.e. respiration, growth efficiency, and carbon demand). Multiple studies observed enhanced rates of extracellular enzyme activity such as leucine-aminopeptidase [10, 12], protease [9], and glucosidase [9, 11–13], suggesting accelerated hydrolysis of various components of dissolved organic material as a function of increasing *p*CO_2_. These studies provide valuable insight to the net community effects of *p*CO_2_ on organic carbon cycling. However, the effects of *p*CO_2_ on heterotrophic bacterioplankton for these studies were evaluated during nutrient-induced phytoplankton blooms (mesocosm studies) [7–10, 12, 13] or by inoculating pH-manipulated phytoplankton cultures with natural bacterioplankton communities (culture study) [11]. Both of these experimental designs make it difficult to differentiate the direct effects of *p*CO_2_ on heterotrophic bacterioplankton physiology and organic carbon removal from the indirect effects that *p*CO_2_ may have had on the quantity and quality of the organic carbon produced by the phytoplankton [14, 15], which could potentially also alter microbial organic carbon removal.

Studies conducted in the absence of phytoplankton showed somewhat different results: Yamada and Suzumura [16] observed no effect of elevated *p*CO_2_ on extracellular glucosidase activity of the free-living bacterioplankton communities. They did, however, observe an increase in extracellular leucine-aminopeptidase and lipase activity, suggesting an increase in the processing of protein and lipid substrates and no effect on polysaccharide use. Siu et al. [17] observed a decrease in bacterioplankton respiration and bacterioplankton production, as measured via ^3^H –thymidine incorporation, and a clear shift from more diverse bacterioplankton communities at ambient pH to less diverse communities under elevated *p*CO_2_ conditions. These studies provide insight to the direct effects of elevated *p*CO_2_ on bacterioplankton extracellular enzyme activity and community structure but did not address the effects of *p*CO_2_ on bacterioplankton removal of organic carbon.

Here we present results from seawater culture experiments that were designed to examine the direct effect of *p*CO_2_ on net removal of organic carbon by bacterioplankton and discuss resulting implications for bacterioplankton respiration and biomass production. Laboratory perturbation experiments using natural bacterioplankton communities were conducted at three contrasting sites: the Sargasso Sea, the Santa Barbara Channel, and the South Pacific Subtropical Gyre. In the oligotrophic Sargasso Sea, deeper waters with elevated *p*CO_2_ are brought to the surface through convective mixing annually [18, 19]. Coastal upwelling off the west coast of North America can induce higher-frequency variability in *p*CO_2_ [20] and result in strong shifts in pH on timescales of days in places like the Santa Barbara Channel, CA [21]. In contrast, seasonal or higher-frequency pulses of elevated *p*CO_2_ are less likely in the more permanently stratified oligotrophic systems such as the waters surrounding the islands of Moorea and Tahiti, within the South Pacific Subtropical Gyre [21]. All ocean surface waters are also subject to rising atmospheric CO_2_ concentrations and are expected to be impacted gradually. How this broad range in the frequency and magnitude of elevated *p*CO_2_ exposure impacts DOC processing by bacterioplankton remains largely unknown. The objective of this study was to assess the direct effect of short-term exposure to elevated *p*CO_2_ on bacterioplankton organic carbon removal, across a variety of ocean sites.

Our results show that short-term perturbations of elevated *p*CO_2_ can result in an increased rate and magnitude of organic carbon removal by bacterioplankton, independent of the environmental origin of the bacterioplankton communities. These results suggest that short-term increases in *p*CO_2_ can lead to an increased loss of organic carbon due to respiration, which may ultimately reduce surface ocean carbon inventories and the amount of DOC available for vertical export from the surface ocean.

## Materials and methods

### General experimental set-up and design

The same general approach was used for all three study sites. Experiments consisted of 0.2 μm-filtered (0.2 μm GSWP, Millipore, Billerica, MA) seawater or 0.2 μm-filtered phytoplankton exudate that was inoculated with natural bacterial communities. The inoculum of natural bacterial communities consisted of either unfiltered whole seawater (Sargasso Sea and South Pacific Subtropical Gyre experiments) or 1.2 μm filtrate (Santa Barbara Channel experiments; 1.2 μm RAWP, Millipore, Billerica, MA). Particulate organic carbon concentration in oligotrophic gyres is low (1–3 μmol C L^-1^) so to avoid filtration artifacts such as reduced bacterial production (unpublished data) and contamination of DOC due to handling, the inoculum was not pre-filtered for the experiments conducted in oligotrophic waters. Because particulate organic carbon concentration can be much greater in coastal upwelling systems it was necessary to remove large particles and organisms from the inoculum. Inoculum was added at 25–30% of final volume (Table 1), effectively diluting grazer concentrations and grazing pressure. All filters were pre-rinsed with ~2 L of deionized distilled water and sample water prior to use in order to remove organic contaminants from the filters.

**Table 1:** Summary of experimental conditions and results.

		Conditions at the Start of the Experiment								Incubation Times		Results		
Exp #	Exp	DOC Source	Volume of Incubation (L)	Ratio of 0.2μm: InoculumSW	T (°C)	Target*p*CO_2_ (ppm)	Actual *p*CO_2_ (ppm)	In situ TOC (μmol C L^-1^)	InitialTOC (μmol C L^-1^)	Time from T_0_ to Stationary-Growth (Days)	Durationof Exp (Days)	TOC Removal (μmol C L^-1^) From T_0_ to Stationary-Growth	TOC Removal (μmol C L^-1^) Over Duration of Exp	BGE (%)
1A	SS Sept. 2012	Natural DOC	2	3:7	26.5	400	~389	73.1 ± 1.3	76.0 ± 0.2	2.13	6.7	ND	ND	ND
1A	–	–	–	–	–	800	~760	–	77.9 ± 0.8	2.13	–	ND	ND	ND
1B	SS Sept. 2012	CNP	2	3:7	26.5	400	~389	73.1 ± 1.3	87.3 ± 1.3	2.13	6.7	-6.7 ± 2.6	-10.2 ± 2.3	11.9 ± 4.9
1B	–	–	–	–	–	800	~760	–	89.5 ± 1.5	2.13	–	-9.2 ± 1.3	-10.6 ± 1.1	9.1 ± 1.3
2A	SBC Dec. 2012	Natural DOC	2	3:7	14.5	250	349 ± 10	85.4 ± 2.0	86.4 ± 0.1	1.34	3.6	-1.7 ± 0.7	-2.0 ± 1.9	40.5 ± 9.4
2A	–	–	–	–	–	1000	1064 ± 10	–	86.1 ± 0.3	1.34	–	-3.8 ± 1.2	-4.7 ± 0.1	30.3 ± 7.2
2B	SBC Dec. 2012	CNP	2	3:7	14.5	250	349 ± 10	85.5 ± 0.5	93.8 ± 0.8	1.34	3.6	-5.8 ± 1.5	-12.0 ± 1.1	35.7 ± 6.0
2B	–	–	–	–	–	1000	1064 ± 10	–	96.5 ± 0.3	1.34	–	-10.7 ± 0.8	-15.1 ± 0.3	26.4 ± 2.1
3	SBC May 2013	*D*. *frag*. Exudate	0.5	1:3	12	250	238 ± 2	88.0 ± 0.1	398.6 ± 3.4	2.75	8	-65.5 ± 1.6	-115.6 ± 3.9	9.1 ± 2.5
3	–	–	–	–	–	1500	1376 ± 45	–	389.6 ± 9.8	2.75	–	-69.9 ± 10.3	-119.2 ± 12.4	6.8 ± 2.8
4	SBC August 2013	*T*. *weiss*. Exudate	2	1:3	14	250	132 ±3	64.2 ± 1.6	157.4 ± 0.9	5.11	6.8	-3.2 ± 0.8	-2.4 ± 0.5	47.0 ± 2.3
4	–	–	–	–	–	1500	1262 ± 6	–	158.2 ± 0.1	5.11	–	-4.9 ± 0.6	-6.52 ± 1.0	36.4 ± 0.3
5A	SBC Oct. 2013	*T*. *weiss*. Exudate	0.5	1:3	16	250	233 ± 3	73.1	155.9 ± 0.3	4.42	12.9	-10.5 ± 0.3	-22.9 ± 3.7	35.6 ± 0.3
5A	–	–	–	–	–	1500	1563 ± 74	–	156.3 ± 0.1	4.42	–	-17.2 ± 0.5	-32.5 ± 13.9	27.1 ± 5.5
5B	SBC Oct. 2013	*C*. *soc*. Exudate	0.5	1:3	16	250	244 ± 5	73.1	141.2 ± 3.2	3.0	12.9	-4.0 ± 0.1	-24.1 ± 4.4	26.1 ± 1.1
5B	–	–	–	–	–	1500	1551 ± 10	–	140.0 ± 2.3	3.0	–	-8.0 ± 4.0	-29.9 ± 4.8	15.7 ± 5.8
5C	SBC Oct. 2013	*A*. *glac*. Exudate	0.5	1:3	16	250	194 ± 10	73.1	142.9 ± 1.5	4.42	12.9	-2.7 ± 2.3	-21.5 ± 3.1	38.4
5C	–	–	–	–	–	1500	1363 ± 5	–	143.5 ± 0.9	4.42	–	-10.2 ± 0.5	-25.2 ± 0.7	31.3 ± 2.9
6	SBC May 2014	*E*. *hux*. Exudate	2	1:3	14.5	250	325 ± 23	ND	103.8 ± 0.1	4.92	14.7	-2.1 ± 1.7	-7.4 ± 1.3	58.4 ± 22.9
6	–	–	–	–	–	1500	1454 ± 14	–	106.9 ± 0.1	5.75	–	-7.5 ± 0.2	-13.5 ± 0.9	28.6 ± 0.6
7A	SPSG July 2014	Natural DOC	2.8	1:3	22	250	ND	70.6	75.4 ± 1.0	ND	10	ND	ND	ND
7A	–	–	–	–	–	1000	ND	–	73.8 ± 0.5	ND	–	ND	ND	ND
7B	SPSG July 2014	*E*. *hux*. Lysate	2.8	1:3	22	250	ND	70.6	85.8 ± 0.6	1.81	10	-4.8 ± 2.3	-11.1 ± 1.1	11.9 ± 5.2
7B	–	–	–	–	–	1000	ND	–	84.4 ± 1.3	1.81	–	-7.0 ± 0.7	-12.4 ± 0.5	8.4 ± 0.7
8A	SPSG July 2014	Natural DOC	2.8	1:3	22	250	ND	76.7	75.2 ± 0.4	ND	10.5	ND	ND	ND
8A	–	–	–	–	–	1000	ND	–	73.7 ± 0.9	ND	–	ND	ND	ND
8B	SPSG July 2014	*E*. *hux*. Lysate	2.8	1:3	22	250	ND	76.7	87.2 ± 0.4	0.92	10.5	-4.7 ± 1.0	-8.0 ± 2.1	24.7 ± 3.0
8B	–	–	–	–	–	1000	ND	–	84.8 ± 0.6	1.84	–	-7.0 ± 0.8	-8.8 ± 2.3	8.5 ± 1.8
9A	SPSG July 2014	Natural DOC	2.8	1:3	22	250	ND	70.9	75.0 ± 2.0	ND	9	ND	ND	ND
9A	–	–	–	–	–	1000	ND	–	75.0 ± 3.6	ND	–	ND	ND	ND
9B	SPSG July 2014	*E*. *hux*. Lysate	2.8	1:3	22	250	ND	70.9	83.7 ± 0.9	1.79	9	-6.7 ± 1.3	-8.9 ± 2.1	8.9 ± 0.7
9B	–	–	–	–	–	1000	ND	–	85.6 ± 0.7	1.79	–	-7.9 ± 0.6	-12.3 ± 0.6	7.3 ± 0.3
10	SBC BC Dec 2015	*T*. *weiss*. Lysate	5	3:7	14	250	ND	69.6	86.4 ± 2.8	2.8	17.5	-15.6 ± 0.1	-19.7 ± 3.0	36.4 ± 0.2
10	–	–	–	–	–	1500	ND	–	1 ± 0.5	2.8	–	-18.0 ± 0.6	-23.2 ± 0.5	34.1 ± 3.7

Conditions at the start of the seawater culture experiments, total organic carbon (TOC) removal from T_0_ to stationary phase, TOC removal over the duration of the incubations, and bacterioplankton growth efficiency (BGE). Mean BGE was calculated as the ratio of the change in the carbon content of bacterioplankton biomass (BC) to dissolved organic carbon (DOC) removal from T_0_ to stationary phase. DOC was calculated as TOC—BC. Bacterioplankton cell abundance was converted to BC carbon using a carbon conversion factor of 30 fg C cell^-1^ for the Santa Barbara Channel (SBC) experiments and 10 fg C cell^-1^ for the experiments conducted in the Sargasso Sea (SS) and South Pacific Subtropical Gyre (SPSG). Error refers to mean ± standard deviation and ‘ND’ refers to not determined. Only one value is recorded in the table when only one valid result was obtained. Dashes refer to the information in the cell above the dash.

#### Treatments

The four types of DOC treatments used in the experiments included either: (1) unamended seawater, which provided naturally occurring DOC, (2) naturally occurring DOC amended with glucose (~10 μM C), NH_4_ Cl (1μM) and K_2_HPO_4_ (0.1μM) (CNP) [22], or one of two phytoplankton-derived DOC mixtures: (3) phytoplankton exudate or (4) naturally occurring DOC amended with phytoplankton lysate (~10 μM C). There was no attempt to standardize the initial concentration of DOC in the phytoplankton exudate treatments; thus, those treatments contained total organic carbon concentrations that were 2–3 fold greater than those in the unamended seawater at the beginning of the experiments (Table 1).

The various treatments were generated by inoculating the 0.2 μm pre-filtered seawater or exudate with the microbial community; this solution was then divided into two polycarbonate (PC) containers to adjust *p*CO_2_ (Table 1). *p*CO_2_ levels were adjusted via chemical additions (Sargasso Sea experiment) or by bubbling with CO_2_-mixed air (Santa Barbara Channel and South Pacific Subtropical Gyre experiments). Adjusted seawater incubations were then transferred into new PC carboys and CNP or lysate was added, if appropriate. A very small volume of lysate (1.2 mL to 11.5 L of experimental volume) or CNP (12 mL to 10 L of experimental water for the Sargasso Sea experiment; 0.28 mL to 10 L of experimental volume for the Santa Barbara Channel experiment) was added to minimize perturbing the carbonate chemistry. All experiments were conducted in duplicate, at in situ temperatures, and in the dark to eliminate photoautotrophic production (Table 1). All PC bottles had been acid-washed (5% or 10% HCL) and rinsed with deionized distilled water and sample water before use.

#### *p*CO_2_ adjustments

Except in the Sargasso Sea, the *p*CO_2_ was adjusted by bubbling with CO_2_-mixed air (Scott Marrin Inc.) at ~100 mL min^-1^ through an air stone to produce fine bubbles for 45 min– 4 hours depending on volume. The *p*CO_2_ adjustment via bubbling was conducted only once, prior to the start of the experiment, to eliminate the possibility of abiotic removal of organic matter as a result of continued bubbling. The air in the headspace of each sample bottle was exchanged with target CO_2_-mixed air every 24 hours to minimize the change in *p*CO_2_ levels throughout the experiment. Bubbling was conducted at experimental temperatures, which were identical to the in situ temperatures of the inoculums. In the Sargasso Sea experiment, *p*CO_2_ was adjusted through the addition of 0.206 g CaCO_3_, 0.032 g NaHCO_3_, and 29 mL of 0.1 N HCL according to the best practices guide [23]. This closed system approach results in an inorganic carbon perturbation that is chemically identical to bubbling [24].

To maintain *p*CO_2_ over the course of the experiments, the incubation bottle lids used for the Santa Barbara Channel and South Pacific Subtropical Gyre experiments were equipped with built-in gas and sampling ports to allow for sampling via positive pressure displacement in which the treatment *p*CO_2_ gas was used to pressurize the head space (< 3 PSI); thus displacing the sample volume from the incubation vessel through the sample line directly into a collection vessel. In the Sargasso Sea, samples were collected by decanting directly from the incubation bottles into sample bottles.

To assess the potential change in pH over the course of an incubation we measured pH as described below for experiments # 1–4. We found that pH changes, within a given incubation, were minimal (0–0.04 pH units) over the course of the experiments. These pH changes correspond to changes in *p*CO_2_ of ~ 1–60 ppm, which is much less than the differences in *p*CO_2_ between low and elevated-*p*CO_2_ treatments (~370 –~1100 ppm). Thus, we concluded that pH adjustments were maintained throughout experiments with very little drift in *p*CO_2_.

#### Sargasso Sea experiment

Water was collected from Hydrostation S (32° 10’N, 64° 30’W) in the northwestern Sargasso Sea at a depth of 10 m using a conductivity, temperature, depth (CTD) rosette. Sampling met the limited impact research requirements under the Bermuda Institute of Sciences Collection and Experimental Ethics Policy. As a result, collection and export permits were not required from the Bermuda Government at the time of this study. Seawater culture experiments were set-up in the lab at the Bermuda Institute of Ocean Science within hours of collection. The experiment consisted of four treatments, each in duplicate: two *p*CO_2_ treatments (ambient and elevated) combined with two DOC treatments (CNP amended and unamended) as a full factorial design (experiment # 1, Table 1). The inoculated seawater was partitioned into two 10 L PC carboys, one remaining unadjusted (ambient *p*CO_2_ treatment); the *p*CO_2_ of the second carboy was adjusted to ~760 ppm using the closed system approach (23). Duplicate 2 L PC bottles were filled with each *p*CO_2_ treatment for the unamended DOC treatments. CNP was added to the seawater remaining in the 10 L carboys to a final concentration of 10 μM C, 1 μM N, and 0.1 μM P (as described above) and each was then split into duplicate 2 L PC bottles, representing the amended DOC treatments. All eight bottles were incubated within a temperature controlled upright incubator (Table 1).

#### Santa Barbara Channel experiments

Five experiments were conducted with surface seawater collected from the Santa Barbara Channel between 2012 and 2014 (experiments # 2–6, Table 1). No specific permissions were required for near-shore seawater collection in the Santa Barbara Channel. Each time, surface seawater was collected using a PC carboy from a near-shore site along the coast of Santa Barbara, CA (34° 24’N, 119° 50’W). The DOC source and starting *p*CO_2_ conditions, as well as the bacterioplankton inoculum varied between experiments (Table 1). *p*CO_2_ of seawater incubations were adjusted to pre-industrial (250 ppm) and elevated (1000 ppm or 1500 ppm) levels by bubbling with CO_2_-mixed air in two 8 L or 20 L PC carboys (see above) depending on experimental water requirements (Table 1). *p*CO_2_ adjusted seawater was split into duplicate 0.5 L or 2 L PC bottles and placed within a temperature controlled upright incubator (Table 1).

#### South Pacific Subtropical Gyre experiments

Three separate experiments were conducted in July 2014 with water collected near French Polynesia in the South Pacific Subtropical Gyre (17° 36’S, 149° 43’W) (experiments # 7–9, Table 1). All research was performed under annual research permits (permit no. 438/AME) issued by the French Polynesian Ministry of Foreign Affairs of International Development of the French Republic, Americans and Caribbean Islands Division. Seawater was collected from a depth of 25 m using a CTD rosette and seawater cultures were prepared in two 20 L PC carboys. Treatments for each of the three experiments included unamended and amended DOC treatments and pre-industrial (250 ppm) and elevated (1000 ppm) *p*CO_2_ in a full factorial design. One of two 20 L carboys was bubbled with pre-industrial (250 ppm) CO_2_-mixed air and the other with elevated (1000 ppm) CO_2_-mixed air as described below. *p*CO_2_ adjusted seawater was then amended with phytoplankton-lysate to a final addition of ~10 μM C and placed within an upright incubator (Table 1). We were unable to measure *p*CO_2_ at sea but applied the same flow rate and bubbling time that resulted in effective *p*CO_2_ adjustment previously.

#### Production of phytoplankton-derived DOC

Phytoplankton exudates and lysates were derived from phytoplankton cultures in order to assess the impact of varying *p*CO_2_ on the microbial consumption of complex DOC. Exudates from five different diatom cultures (*Dactyliosolen fragilissimus*, *Thalassiosira weissflogii*, *Chaetoceros socialis*, *Asterionellopsis glacialis*) and one coccolithophore (*Emiliana huxleyi*) were used in the Santa Barbara Channel experiments where diatoms are often abundant. In addition, phytoplankton lysate was produced from *Emiliana huxleyi* and used for the experiments conducted in the South Pacific Subtropical Gyre where coccolithophores are often present [25].

Phytoplankton cultures were grown in sterilized (double 0.2 μm filtered) seawater collected from the Santa Barbara Channel and enriched with inorganic nutrients in a modified version of f/2 medium [26]. Preliminary growth experiments were used to determine the ratio of N:P:Si nutrient additions needed to induce nitrate limitation within each culture. This was done to enhance the production of DOC in the phytoplankton cultures and to minimize the addition of nitrate to the culture experiments. A light/ dark cycle of 14/10 hours, a photon flux density of ~100 μmol m^-2^ s^-1^, and a temperature of 18°C was maintained for all cultures throughout the growth period.

Nitrate concentration was monitored over the course of phytoplankton growth via UV detection using an HP452A spectrophotometer (Hewlett Packard 8452A) [27] and calibrated against a series of chemical nitrate standards (4 point curve; 0–100 μmol N L^-1^). Exudate and lysate was harvested from the cultures two days after nitrate concentration fell below 2.4 ± 1 μmol N L^-1^ according to Nelson and Carlson [28]. Briefly, exudate was harvested via filtration (0.2 μm GSWP, Millipore, Billerica, MA), whereas lysate was harvested through a series of steps including cell concentration via centrifugation (10,000 rpm) and freeze-thaw cycles. After final centrifugation the cell pellet was abraded with a pre-combusted glass rod to generate cell lysate. Final lysate volume was 0.2 μm filtered and then acidified (4 M HCl) to a pH of ~3 and stored at -20°C for one week prior to use in the South Pacific Subtropical Gyre experiments.

#### Experimental samples

Experimental samples were not filtered upon removal from experimental incubations in order to minimize contamination due to transfer and handling. Samples for total organic carbon (TOC; carbon content of bacterioplankton biomass plus DOC) and bacterioplankton abundance were collected throughout the incubations. pH was monitored over regular intervals for the Sargasso Sea experiments and experiments # 2–5 in the Santa Barbara Channel. *p*CO_2_ was measured at the start of the incubations for Santa Barbara Channel experiments # 2 and 4–6. Measurements of *p*CO_2_ and pH were not made for the South Pacific Subtropical Gyre experiments or the Santa Barbara Channel #10 experiment due to logistical constraints. However, our ability to effectively alter the *p*CO_2_ from ambient to target levels was demonstrated numerous times prior to these experiments (Table 1, experiments # 2–6). Thus, we used previously determined bubbling rates and duration to achieve target *p*CO_2_ levels. The direct effect of *p*CO_2_ on carbon content of bacterioplankton cells was determined during the Santa Barbara Channel follow-up experiment # 10.

### Sample processing

TOC measurements—The procedures used to set up each experiment (inoculum filtration and dilution with 0.2 μm filtrate) removed the majority of particulate organic carbon such that changes in bacterioplankton carbon production and DOC removal were mainly a function of the growth of the bacterioplankton. Ideally, samples collected for organic carbon would be filtered in order to directly assess DOC removal independently from bacterioplankton carbon production. However, sample handling during filtration can result in contamination that obscures changes in DOC on the scale of a few micro-molar C. To avoid contamination, seawater samples from the incubation experiments were not filtered. Therefore, measured values of organic carbon include both DOC and bacterioplankton carbon and are considered TOC.

TOC samples were collected into 60 mL high-density polyethylene bottles (Sargasso Sea and South Pacific Subtropical Gyre) or in combusted 40 mL glass EPA vials with Teflon coated silicone septa (Santa Barbara Channel). All TOC samples were frozen at -20°C until analysis. Samples were analyzed via high temperature combustion method on a modified Shimadzu TOC-V or Shimadzu TOC-L using the standardization and referencing approaches described in Carlson et al. [29].

Bacterioplankton abundance measurement—Samples for bacterioplankton abundance were analyzed by epifluorescence microscopy with 0, 6-diamidino -2-phenyl dihydrochloride (5μg mL^-1^, DAPI, SIGMA-Aldrich, St. Louis, MO, USA) according to Porter and Feig [30] (experiments # 1 and 10), or by Flow Cytometry (FCM) on an LSR II with SYBR Green I according to Nelson et al. [31] (experiments # 2–9). See Parsons et al. [32] and Nelson et al. [31] regarding sample preparation and instrument settings for epifluorescence microscopy and FCM analyses, respectively. DAPI direct counts and FCM analysis enumerate total prokaryotic abundance. We were not able to differentiate between bacterial and archaeal domains and refer to the combined cell densities as bacterioplankton abundance [33].

Bacterioplankton carbon measurement—To assess the direct effect of *p*CO_2_ on the carbon content of bacterioplankton cells a follow-up experiment was conducted with water collected from the Santa Barbara Channel during December 2015 (experiment # 10, Table 1). The experimental procedure used was identical to that for all other experiments. Surface seawater was collected using a PC carboy from a near-shore site along the coast of Santa Barbara, CA (34° 24’N, 119° 50’W). One of two 20 L carboys filled with 0.2 μm- and 1.2 μm-filtered seawater was bubbled with pre-industrial (250 ppm) CO_2_-mixed air and the other with elevated (1500 ppm) CO_2_-mixed air. *p*CO_2_ adjusted seawater was then amended with phytoplankton-lysate to a final addition of ~20 μmol C L^-1^ and placed within a temperature controlled incubator in the dark (Table 1).

Samples for determining the carbon content of bacterioplankton cells were collected at the beginning of stationary phase to maximize bacterioplankton abundance and to minimize the contribution of nanoflagellate grazers to carbon estimates. A one-liter sample was filtered onto combusted GF75 filters (Advantec^®^; Dublin CA). GF75 filters were used because they have a nominal pore size of 0.3 μm and demonstrated 92 ± 2% (range = 3.7%; n = 5) cell retention. DOC-blanks were collected simultaneously to account for adsorption of organic carbon to the filters and were collected by filtering ~1 L of pre-filtered (0.2 μm) experimental volume onto a 0.3 μm GF75. Filters were placed into pre-combusted (4 hr at 400°C) glass vials, and frozen at -40°C. Filters were shipped on dry ice to Bigelow Analytics (Bigelow Laboratories for Ocean Sciences) and particulate organic carbon was determined using a Costech ECS 4010 elemental analyzer (980° combustion). Filters were analyzed within two weeks of collection and DOC-blank values were subtracted from experimental particulate organic carbon values. Carbon content of bacterioplankton cells (i.e. the carbon conversion factor) was calculated as particulate organic carbon concentration divided by bacterioplankton abundance.

#### Carbonate parameters

pH- Spectrophotometric pH measurements were made via absorbance of seawater sample at 25°C with m-cresol purple indicator dye (unpurified) following Dickson et al. [34] for the Santa Barbara Channel experiments. The dye was frequently calibrated against certified reference material (Batch # 10, Dickson, La Jolla, California). pH was measured using a Corning 430 pH meter for the Sargasso Sea experiments.

*p*CO_2_ –Samples for *p*CO_2_ analysis were collected into ~400 mL borosilicate bottles according to Dickson et al. [34]. The bottles were capped immediately upon collection and *p*CO_2_ analysis was conducted within 24 hrs of sampling on a custom *p*CO_2_ system according to Hales et al. [35]. All *p*CO_2_ samples were systematically referenced against standard quality gasses (Scott Marrin Inc.).

Measurements of carbonate parameters were not made for the South Pacific Subtropical Gyre and Santa Barbara Channel # 10 experiments due to logistical constraints. We used bubbling flow rates and duration determined from previous experimental volumes to achieve target elevated and pre-industrial *p*CO_2_ levels for these experiments. For all other experiments where *p*CO_2_ was not directly measured (experiments # 1 and 3), it was calculated based on pH and a recent local in situ measurement of total alkalinity using CO2SYS [36] and the dissociation constants for carbonic acid as refitted by Dickson and Millero [37].

### Calculations

Cell-specific TOC removal*—*Cell-specific TOC removal was calculated as the difference in TOC concentration (ΔTOC) from the start of the experiment to the beginning of stationary phase divided by the concomitant change in bacterioplankton abundance.

Bacterioplankton carbon demand*—*Bacterioplankton carbon demand (BCD) is equivalent to the carbon required for bacterioplankton biomass production (ΔBC) plus bacterioplankton respiration (BR; BCD = ΔBC + BR), over a given period of time. BCD is also equivalent to the total amount of DOC consumed by heterotrophic microbial communities. Therefore, bacterioplankton carbon demand was calculated as the change in the concentration of DOC from the start of the incubations to stationary phase. DOC concentration was calculated as the difference between the concentrations of TOC and BC (i.e. DOC = TOC—BC).

Bacterioplankton growth efficiency—Bacterioplankton growth efficiency, which provides an estimate of how much of the consumed organic carbon is partitioned into cellular biomass (BC) versus respiration [38, 39], was calculated as the increase in BC from the start of the incubations to stationary growth phase divided by bacterioplankton carbon demand. For the Santa Barbara Channel experiments, bacterioplankton abundance was converted to BC using the carbon conversion factor of 30 fgC cell^-1^ measured during Santa Barbara Channel experiment # 10. This is comparable to other published carbon conversion factors ([40], A Cano, unpublished). A lower conversion factor of 10 fgC cell^-1^ was used for communities from the Sargasso Sea and the South Pacific Subtropical Gyre to reflect smaller cells in oligotrophic regions [41, 42].

Bacterioplankton respiration—Bacterioplankton respiration can be equated to the change in ΔTOC by the following argument. The change in TOC between time 1 and time 2 is given by:

$$\Delta TOC\;=\;\left(DOC_{1}\;+\;BC_{1}\right)\;–\;\left(DOC_{2}\;+\;BC_{2}\right)$$ (1)

Then because bacterioplankton grow by consuming DOC, converting a portion of consumed DOC into BC and respiring the remainder to CO_2_, the change in DOC between two time points (ΔDOC) is given by:

$$\Delta DOC\;=\;\left(BC_{2}\;–\;BC_{1}\right)+\;BR$$ (2)

Note that in this formulation ΔDOC is a positive value such that when Eq 1 is rearranged as:

$$\Delta TOC\;=\;\left(DOC_{1}\;–\;DOC_{2}\right)+\;\left(BC_{1}–\;BC_{2}\right)$$ (3)

the right hand side of Eq 2 can be substituted for (DOC_1_ –DOC_2_) in Eq 3 yielding:

$$\Delta TOC\;=\;\left(BC_{2}\;–\;BC_{1}\right)\;+\;BR\;+\;\left(BC_{1}\;–\;BC_{2}\right)\;=\;BR$$ (4)

## Results

**Fig 1:**
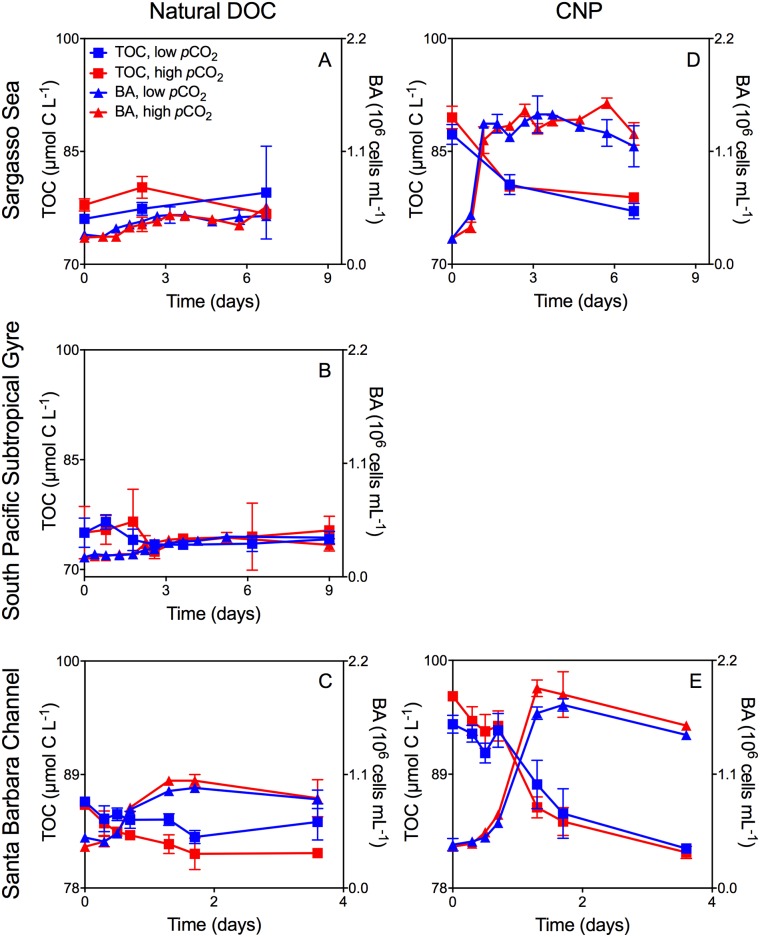
Mean TOC concentration and mean bacterioplankton abundance for Sargasso Sea and Santa Barbara Channel CNP- and natural-DOC experiments. Mean TOC concentration (± SD) and mean bacterioplankton abundance (BA; ± SD) averaged across duplicate incubations through time and as a function of *p*CO_2_ for natural bacterioplankton assemblages incubated with: A: Natural DOC, Sargasso Sea Exp. #1A; B: Natural DOC, South Pacific Subtropical Gyre Exp. #7; C: Natural DOC, Santa Barbara Channel Exp. #2A; D. CNP, Sargasso Sea Exp. #1B; E. CNP, Santa Barbara Channel Exp. #2B. For treatments at all sites, red denotes elevated *p*CO_2,_ while blue denotes ambient *p*CO_2_ in the Sargasso Sea experiment and pre-industrial *p*CO_2_ in the Santa Barbara Channel and South Pacific Subtropical Gyre experiments. Squares represent TOC (μmol C L^-1^) while triangles denote BA (10^6^ cells mL^-1^) over the course of the incubation.

**Fig 2:**
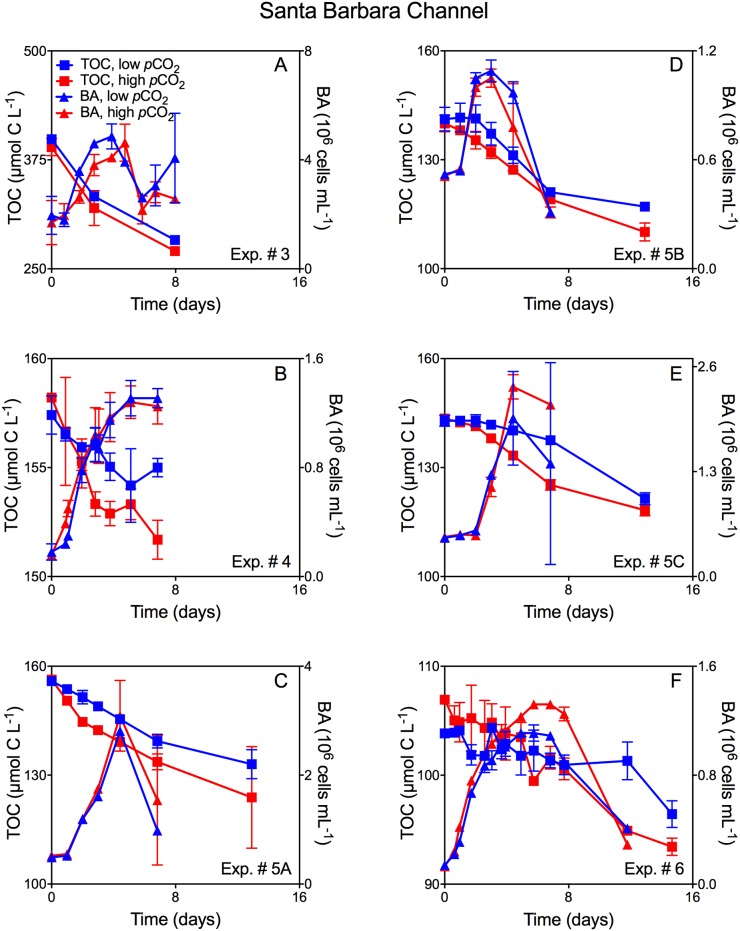
Mean TOC concentration and mean bacterioplankton abundance for Santa Barbara Channel phytoplankton-DOC experiments. Mean TOC concentration (± SD) and mean bacterioplankton abundance (BA; ± SD) averaged across duplicate incubations through time and as a function of *p*CO_2_ for natural bacterioplankton assemblages in the Santa Barbara Channel experiments incubated with: A: *D*. *fragilissimus*-derived DOC, Exp. #3; B: *T*. *weissflogii*-derived DOC, Exp. #4; C: *T*. *weissflogii*-derived DOC, Exp. #5A; D: *C*. *socialis-*derived DOC, Exp. #5B; E: *A*. *glacialis*-derived DOC, Exp. #5C; F: *E*. *huxleyi*-derived DOC, Exp. #6. Red denotes elevated *p*CO_2,_ while blue denotes low (250 ppm) *p*CO_2_. Squares represent TOC (μmol C L^-1^) while triangles denote bacterioplankton abundance (10^6^ cells mL^-1^) over the course of the incubation.

**Fig 3:**
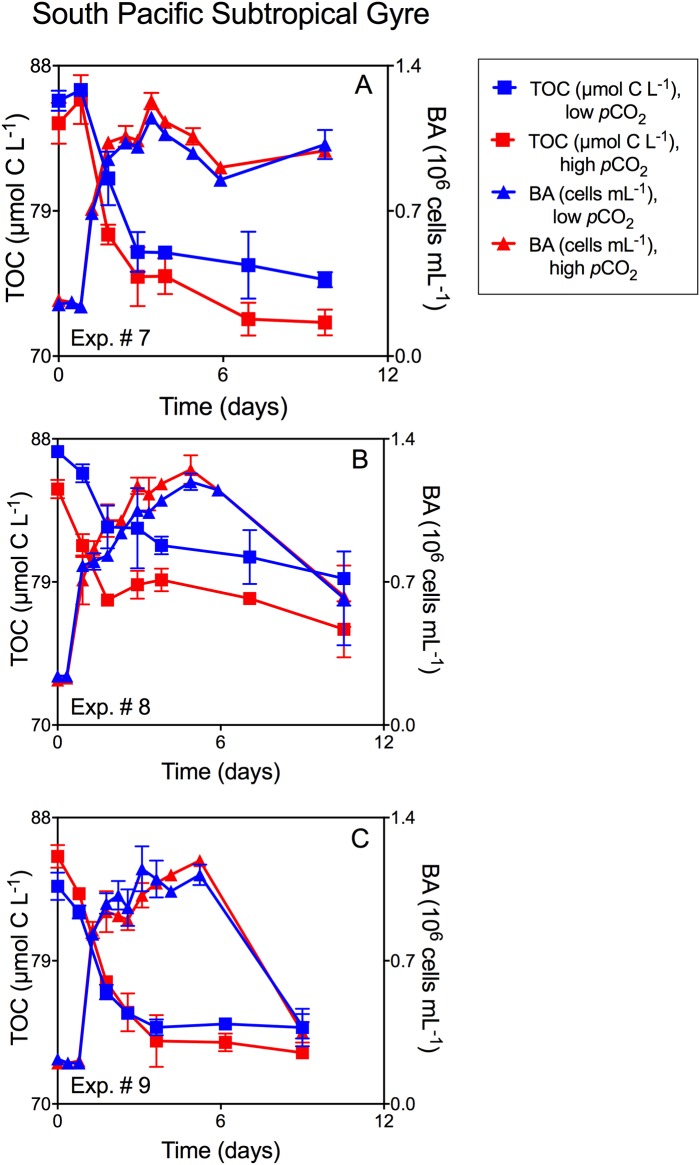
Mean TOC concentration and mean bacterioplankton abundance for phytoplankton-DOC South Pacific Subtropical Gyre experiments. Mean TOC concentration (± SD) and mean bacterial abundance (BA; ± SD) averaged across duplicate incubations through time and as a function of *p*CO_2_ for natural bacterioplankton assemblages in the South Pacific Subtropical Gyre experiments incubated with *E*. *huxleyi*-derived DOC: A: Exp. #7B; B: Exp. #8B; C: Exp. #9B. The same experimental design was used for these experiments (note that only the amended treatments are shown). Red denotes elevated *p*CO_2,_ while blue denotes low (250 ppm) *p*CO_2_. Squares represent TOC (μmol C L^-1^) while triangles denote BA (10^6^ cells mL^-1^) over the course of the incubation.

Growth patterns of the natural bacterioplankton communities within all experiments demonstrated typical batch culture patterns of lag, exponential, and stationary growth (Figs 1, 2, and 3). However, the magnitude of the response differed between sites and treatments as described below.

### Experiments with naturally occurring DOC

Bacterioplankton communities in both elevated and pre-industrial *p*CO_2_ treatments, grown on naturally occurring DOC in the Sargasso Sea, demonstrated a less than two-fold change in bacterioplankton abundance and no resolvable removal of TOC over the course of the incubations (Fig 1A). *p*CO_2_ treatments showed no difference in cell yield (4.8 ± 0.8 x10^5^ cells mL^-1^; elevated *p*CO_2_ vs. 4.8 ± 0.3 x10^5^ cells mL^-1^; ambient *p*CO_2_) or production rate (6.0 ± 2.2 x10^4^ cells mL^-1^ d^-1^; elevated *p*CO_2_ vs. 7.0 ± 1.4 x10^4^ cells mL^-1^ d^-1^; ambient *p*CO_2_) (Fig 1A). A similar pattern was detected in the South Pacific Subtropical Gyre for the unamended experimental treatments (Fig 1B).

**Fig 4:**
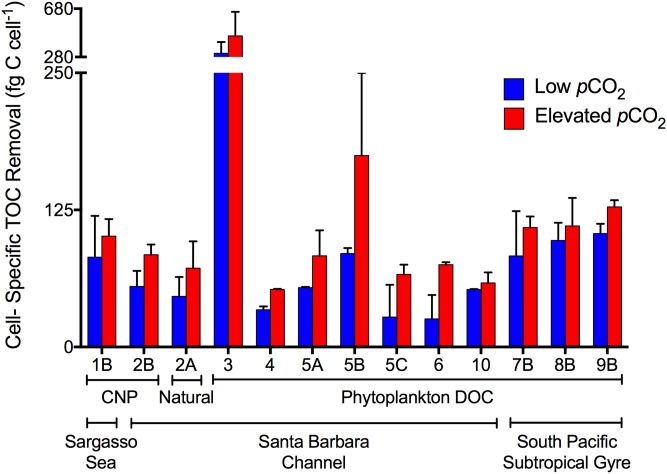
Cell-specific TOC removal. Mean (± SD) cell-specific TOC removal averaged across duplicate incubations and calculated as the change in TOC divided by the concurrent change in bacterioplankton abundance (BA) from the start of the incubations to stationary phase. Numbers on X-axis label refer to the experiment number (Table 1). Colors denote cell-specific TOC removal at elevated *p*CO_2_ (red) and low (ambient or pre-industrial) *p*CO_2_ (blue). Only experiments where a change in TOC was resolvable are shown.

In contrast, the experiment with naturally occurring DOC from the Santa Barbara Channel demonstrated significant increases in bacterioplankton abundance and measurable removal of TOC in both pre-industrial and elevated *p*CO_2_ treatments (Fig 1C). Although the initial rate of increase in bacterioplankton abundance was similar for both *p*CO_2_ treatments, greater maximum abundance was obtained by stationary phase with elevated *p*CO_2_ (1.0 ± 0.02 x10^6^ cells mL^-1^), compared with pre-industrial (250 ppm) *p*CO_2_ levels (0.9 ± 0.01 x10^6^ cells mL^-1^) (Fig 1C). The rate of TOC removal was also greater in the elevated *p*CO_2_ treatment (-2.8 ± 0.9 μmol C L^-1^ d^-1^) relative to the pre-industrial *p*CO_2_ treatment (-1.3 ± 0.5 μmol C L^-1^ d^-1^) (Fig 1C). The enhanced TOC removal relative to the corresponding change in bacterioplankton abundance led to greater cell-specific consumption of TOC at elevated *p*CO_2_ (71.9 ± 24.4 fg C cell^-1^) relative to pre-industrial *p*CO_2_ (46.1 ± 17.7 fg C cell^-1^; Fig 4).

### Experiments with CNP

The addition of glucose in the CNP- Sargasso Sea experiment raised TOC concentration by 11.2 and 11.6 μmol C L^-1^ over the unamended treatment for the ambient and elevated *p*CO_2_ treatments, respectively. This additional labile organic carbon (in combination with ammonium and phosphate) enhanced overall bacterioplankton yield by greater than an order of magnitude (Fig 1D) compared to the unamended treatment (Fig 1A). The bacterioplankton communities removed an amount of TOC equal to those carbon amendments in both elevated and ambient *p*CO_2_ treatments implying that throughout the incubation little, if any, of the naturally occurring DOC was consumed, e.g. no priming effect was observed. However, TOC removal rates during exponential growth were greater under elevated *p*CO_2_ compared to ambient *p*CO_2_ (-4.3 ± 0.5 μmol C L^-1^ d^-1^ vs. -3.1 ± 1.2 μmol C L^-1^ d^-1^, respectively; Fig 1D). Greater bacterioplankton abundance at elevated *p*CO_2_ (1.4 ± 0.02 x10^6^ cells mL^-1^) relative to ambient *p*CO_2_ (1.2 ± 0.04 x10^6^ cells mL^-1^) was proportionately smaller than enhanced TOC removal during the same period, resulting in greater cell-specific consumption of TOC at elevated *p*CO_2_ (101.1 ± 15.6 fg C cell^-1^) relative to ambient *p*CO_2_ (81.9 ± 37.7 fg C cell^-1^; Fig 4).

The addition of glucose in the CNP- Santa Barbara Channel experiment raised TOC concentration by 7.5 and 10.5 μmol C L^-1^ over the unamended treatment for the pre-industrial (250 ppm) and elevated (1000 ppm) *p*CO_2_ treatments, respectively. Bacterioplankton abundance increased by over an order of magnitude (Fig 1E) compared to the unamended treatment (Fig 1C). An amount of TOC greater than the amendments was removed in both *p*CO_2_ treatments over the course of the experiment (compare initial TOC in Fig 1C with the final TOC in Fig 1E); however, both total TOC drawdown and the rate of TOC removal during exponential phase were greater under elevated *p*CO_2_ (-8.0 ± 0.6 μmol C L^-1^ d^-1^) relative to the pre-industrial *p*CO_2_ treatment (-4.3 ± 1.1 μmol C L^-1^ d^-1^). The bacterioplankton abundance in stationary phase was also greater at elevated *p*CO_2_ (1.9 ± 0.1 x10^6^ cells mL^-1^) than at pre-industrial *p*CO_2_ (1.7 ± 0.06 x10^6^ cells mL^-1^); however, the enhanced TOC removal relative to the increase in bacterioplankton, again resulted in greater cell-specific TOC consumption at elevated *p*CO_2_ (84.1 ± 9.3 fg C cell^-1^) relative to ambient *p*CO_2_ (55.2 ± 14.2 fg C cell^-1^; Fig 4).

Final TOC concentration in the Santa Barbara Channel experiment was drawn down to the same final concentration with and without CNP in elevated *p*CO_2_ incubations (compare Fig 1C and 1E) indicating that CNP addition did not enhance the consumption of naturally occurring DOC under elevated *p*CO_2_. In contrast, final TOC concentration in the pre-industrial *p*CO_2_ CNP incubations was ~3 μmol C L^-1^ lower than in the unamended incubations (compare Fig 1C and 1E), indicating that the addition of CNP allowed for greater consumption of the naturally occurring DOC (a priming effect) under pre-industrial *p*CO_2_.

### Experiments with phytoplankton-derived DOC

TOC removal rates and the magnitude of TOC removal through stationary growth phase were enhanced in elevated *p*CO_2_ treatments for all experiments with phytoplankton-derived DOC (Figs 2 and 3), indicating greater consumption of the exudates and lysates at elevated *p*CO_2_ compared to pre-industrial levels. While trends in TOC removal were consistent across experiments and DOC sources, the bacterioplankton abundance yield was variable with some treatments having elevated yield in high *p*CO_2_ treatments (Fig 2E and 2F; Fig 3B), and others showing no difference (Fig 1A, 1B, 1C and 1D; Fig 3A and 3C) between *p*CO_2_ treatments. Despite variability in bacterioplankton abundance yield, elevated *p*CO_2_ resulted in TOC removal by bacterioplankton that was always proportionately greater than the corresponding increases in abundance, leading to greater cell-specific removal at elevated *p*CO_2_ (Fig 4).

### Carbon content of bacterioplankton cells

Trends in TOC removal and bacterioplankton abundance in the Santa Barbara Channel experiment # 10 were consistent with all previous Santa Barbara Channel experiments: Greater TOC removal through stationary growth phase was observed in elevated *p*CO_2_ incubations (-18.0 ± 0.6 μmol C L^-1^) relative to pre-industrial *p*CO_2_ incubations (-15.6 ± 0.1 μmol C L^-1^), while bacterioplankton abundance yield was similar across *p*CO_2_ treatments (4.0 ± 0.5 x10^6^ cells mL^-1^; elevated *p*CO_2_ vs. 3.8 ± 0.1 x10^6^ cells mL^-1^; pre-industrial *p*CO_2_). No resolvable difference in the carbon content of bacterioplankton cells was observed between cells as a function of *p*CO_2_ (31.4 ± 1.2 fg C cell^-1^; pre-industrial *p*CO_2_ vs. 31.8 ± 1.8 fg C cell^-1^; elevated *p*CO_2_; p-value > 0.66, t-test).

### Bacterioplankton carbon removal and growth dynamics

**Fig 5:**
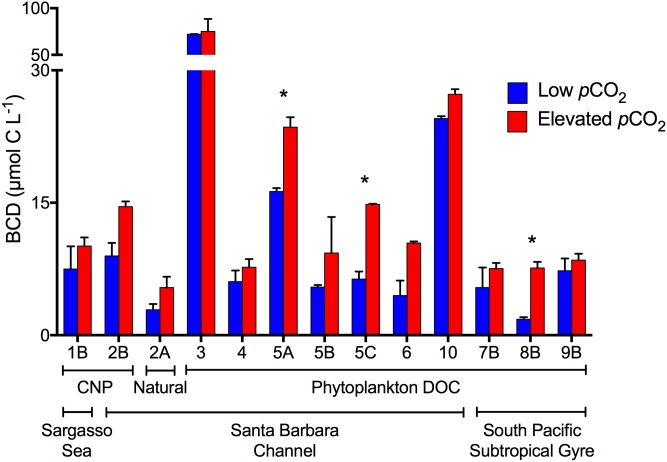
Bacterioplankton Carbon Demand. Mean (± SD) bacterioplankton carbon demand (BCD) averaged across duplicate incubations and calculated as the change in DOC from T_0_ to stationary phase. Change in DOC was calculated as TOC less the carbon content of bacterioplankton biomass (using a carbon conversion factor of 30 fg C cell^-1^ for bacterioplankton in the Santa Barbara Channel and 10 fg C cell^-1^ for bacterioplankton in the Sargasso Sea and South Pacific Subtropical Gyre). Colors denote BCD at elevated *p*CO_2_ (red) and low (ambient or pre-industrial) *p*CO_2_ (blue). Numbers refer to the experiment number (Table 1). Only experiments where a change in TOC was resolvable are shown.

**Fig 6:**
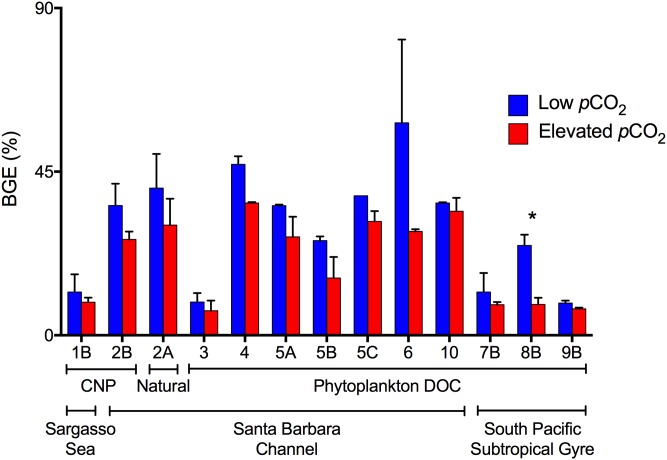
Bacterioplankton growth efficiency. Mean (± SD) Bacterioplankton growth efficiency averaged across duplicate incubations and calculated as the change in bacterioplankton biomass carbon (using a carbon conversion factor of 30 fg C cell^-1^ for bacterioplankton in the Santa Barbara Channel and 10 fg C cell^-1^ for bacterioplankton in the Sargasso Sea and South Pacific Subtropical Gyre) divided by the concurrent change in DOC from T_0_ to stationary phase. Colors denote bacterioplankton growth efficiency at elevated *p*CO_2_ (red) and low (ambient or pre-industrial) *p*CO_2_ (blue). Numbers refer to the experiment number (Table 1). Only experiments where a change in TOC was resolvable are shown.

Bacterioplankton carbon removal and growth dynamics were evaluated for experiments in which TOC removal was measureable. Effects of *p*CO_2_ on carbon removal and bacterioplankton growth dynamics within experiments were tested for significance by the Alexander-Govern test. This first approximation, unequal variance test was used to account for small sample sizes (n = 2 for each *p*CO_2_ level) and, therefore, an inability to test for normality and homoscedasticity with substantial power (p-values are reported in Table 2). Despite the fact that few within experiment comparisons resulted in significant effects of *p*CO_2_, patterns in carbon removal and bacterioplankton growth dynamics were consistent across all experiments and locations, and are highly significant: cell-specific TOC removal (Fig 4) and bacterioplankton carbon demand (Fig 5) were consistently enhanced at elevated *p*CO_2_ (p-value = 0.0003, Fisher’s sign test; p-value < 0.0001, t-test, respectively), while bacterioplankton growth efficiency (Fig 6) was significantly reduced at elevated *p*CO_2_ (p-value = 0.0003, Fisher’s sign test). Significance of these consistent patterns was tested using the consensus t-test or the Fisher’s sign-test, when tests for normality and homoscedasticity failed. Thus, while our measurement precision was inadequate to demonstrate statistically significant differences at any given location, the ecological and biogeochemical significance across all sites is statistically clear in that the consistency of the response shows that an enhancement of cell-specific TOC removal and carbon demand, and a reduction in bacterioplankton growth efficiency, under elevated *p*CO_2_ is a common feature across the wide range of ocean habitats examined.

**Table 2:** P values for bacterioplankton carbon removal and growth dynamics.

Experiment #	Cell-specific TOC removal p-value	BCD p-value	BGE p-value
1B	0.62	0.39	0.57
2A	0.40	0.17	0.40
2B	0.18	0.07	0.25
3	0.52	0.80	0.51
4	0.05	0.33	0.08
5A	0.33	0.03*	0.27
5B	0.34	0.41	0.23
5C	0.30	0.03*	NA
6	0.19	0.12	0.32
7B	0.54	0.41	0.52
8B	0.64	0.02*	0.04*
9B	0.12	0.45	0.15
10	0.54	0.05	0.54

Alexander-Govern approximate p-values, calculated for cell-specific TOC removal, bacterioplankton carbon demand (BCD) and bacterioplankton growth efficiency (BGE). Asterisks denote p-values < 0.05 and ‘ND’ refers to not determined, due to lack of replication.

## Discussion

The results indicate that elevated *p*CO_2_ can directly influence bacterioplankton removal of organic carbon. Elevated *p*CO_2_ led to greater TOC removal by bacterioplankton communities growing on a range of DOC from glucose to more complex DOC, consisting of phytoplankton products or naturally occurring DOC. This result was observed across all ocean regions for experiments where DOC was amended. Similar results were observed in the unamended Santa Barbara Channel incubations at elevated *p*CO_2_ but it was not possible to discern whether *p*CO_2_ affected consumption of naturally occurring DOC in the South Pacific Subtropical Gyre and Sargasso Sea, as changes in TOC were below analytical detection limits in these more oligotrophic environments.

Our results suggest that short-term increases in *p*CO_2_ will lead to enhanced removal of bioavailable surface organic carbon by heterotrophic bacterioplankton communities. Through observations of enhanced extracellular glucosidase activity, previous mesocosm and culture studies suggested that elevated *p*CO_2_ conditions might lead to greater removal of organic carbon [9, 11–13]. Here we show a clear, direct effect of elevated *p*CO_2_ on bacterioplankton-mediated organic carbon removal. Both our work and that of the mesocosm and culture studies [9, 11–13] suggest that short-term exposure to elevated *p*CO_2_ will enhance the bacterioplankton removal of bioavailable DOC in the surface ocean.

### Experimental design considerations

In order to investigate the direct effect of *p*CO_2_ on organic carbon consumption by natural bacterioplankton communities, we employed the following in our experimental design: (1) Experiments were conducted in the dark to eliminate photoautotrophic organic carbon production; (2) To ensure that the majority of organic carbon removal could be attributed to heterotrophic bacteria, we used seawater culture dilution methods [43] to dilute and minimize the effects of protistan grazer populations [44, 45, 46]; (3) To further minimize the potential effects of grazers on organic carbon consumption, calculations were made from the start of the incubations to stationary growth phase, ensuring low grazer densities and high bacterial densities during the period over which bacterioplankton physiological parameters were estimated. However, we cannot rule out the possibility that groups other than heterotrophic bacteria (e.g. archaea and marine protists) contributed to organic carbon removal in these experiments because we were unable to resolve changes in their abundance. The consumption of specific organic compounds (i.e. amino acids) by other members of marine plankton has been observed [47–49] but their effect on removal of bulk dissolved organic material remains largely unquantified [50].

To minimize the potential for abiotic removal of organic carbon (e.g. via adhesion to the incubation-bottle walls or accumulation at the air-water interface) our seawater cultures were only bubbled before the start of the experiments (before T_0_). *p*CO_2_ levels were maintained over the course of the experiments through the use of gas-tight sampling ports (see methods) and by replacing the headspace with target *p*CO_2_ –gas, daily. We cannot rule out the possibility that abiotic removal of organic carbon occurred during bubbling; however, continued abiotic removal over the course of the experiment did not occur as is evidenced by the fact that no resolvable removal of TOC was detected in experiments in which bacterioplankton growth was minimal (Fig 1A and 1B, Table 1: experiments #1A, and 7A, 8A, and 9A).

We attribute organic carbon removal (ΔTOC) to consumption by heterotrophic bacterioplankton in our experiments. The filtration and dilution steps used in our experimental set-up were designed to minimize or eliminate the contribution of non-living particulate organic carbon in experimental incubations. That means that a measurement of TOC represents the sum of DOC and the carbon content of bacterioplankton biomass. It also means that the change in TOC (ΔTOC) is a measure of the amount of carbon lost to bacterioplankton respiration by the argument described in the materials and methods section (see *bacterioplankton respiration* calculation). This tight coupling between the direct measurement of ΔTOC and the production of CO_2_ (i.e. bacterioplankton respiration) was shown empirically in similar seawater culture experiments, supporting the use of ΔTOC as a proxy for heterotrophic bacterioplankton respiration in this type of experiment [45].

### Bacterioplankton removal of organic carbon

Highly productive systems like the coastal upwelling regions of the Santa Barbara Channel can create food-webs that allow for greater DOC production than heterotrophic bacterioplankton consumption, resulting in the accumulation of bioavailable DOC [51, 52, 40]. In the present study we show enhanced removal of naturally occurring organic carbon by Santa Barbara Channel heterotrophic bacterioplankton communities under increased *p*CO_2_, suggesting that rapid increases in *p*CO_2_, common in upwelling systems, can accelerate the consumption of DOC in the surface ocean.

In contrast, removal of TOC in the naturally occurring DOC treatments conducted in the Sargasso Sea and the South Pacific Subtropical Gyre was below limits of detection, indicating that only a very small fraction of the naturally occurring DOC was available to the extant bacterioplankton on the time-scale of these experiments. This result is consistent with previous observations indicating that because DOC production and consumption processes in the oligotrophic gyres are so tightly coupled little, if any, of the DOC that accumulates in these regions is bioavailable to surface bacterioplankton communities on the time scale of hours to days [44, 53].

To assess the effect of *p*CO_2_ on the removal of recently produced DOC in systems where DOC use is difficult to resolve, we simulated food-web production of complex DOC by adding phytoplankton-derived DOC to seawater culture experiments. These amendment experiments were conducted in both coastal (Santa Barbara Channel) and open ocean systems (South Pacific Subtropical Gyre) and show a consistent pattern of an increased magnitude of TOC removal with elevated *p*CO_2_. While these results show clear short-term trends regarding the removal of phytoplankton-derived DOC, longer-term experiments (weeks to months) must be conducted to properly evaluate whether exposure to elevated *p*CO_2_ sustains an offset in the magnitude of TOC removal compared to ambient and pre-industrial *p*CO_2_ conditions.

Collectively, our experiments indicate that short-term increases in *p*CO_2_ directly influence bacterioplankton removal of organic carbon. Elevated *p*CO_2_ led to greater TOC removal by natural bacterioplankton communities growing on a range of organic carbon compounds from glucose, to more complex phytoplankton products, to naturally occurring DOC. Assuming that heterotrophic bacterioplankton are driving the removal of TOC, then these results suggest that short-term exposure to elevated *p*CO_2_ leads to enhanced bacterioplankton respiration relative to pre-industrial *p*CO_2_. Enhanced respiration under elevated *p*CO_2_ coupled with minimal change in bacterioplankton abundance yielded systematically greater cell-specific respiration (i.e. cell-specific TOC removal) in elevated *p*CO_2_ treatments (Fig 4). Even in cases where bacterioplankton yield was greater under elevated *p*CO_2_, that effect was outweighed by enhanced respiration leading to greater amounts of carbon respired per cell. This result suggests that during periods of acidified conditions, more bioavailable organic carbon in surface waters will be converted to CO_2_, decreasing the amount of organic carbon available for potential export and increasing the likelihood that carbon will be lost from the surface ocean to the atmosphere through outgassing of CO_2_.

### Bacterioplankton growth dynamics

Organic carbon removal was tracked by measuring TOC; as a result, these seawater experiments do not directly address the effect of *p*CO_2_ on BC production or DOC consumption. However, these components were evaluated using the direct estimates of the carbon content of bacterioplankton cells from the December 2015 Santa Barbara Channel experiment. Knowledge of the carbon content of bacterioplankton cells enables the calculation of BC to be easily extrapolated from cell abundance data and also enables DOC to be calculated as the difference between TOC and BC. Estimates of BC and DOC removal can then be used to inform aspects of bacterioplankton physiology by providing estimates of bacterioplankton carbon demand (i.e. DOC consumption) and the fraction of consumed DOC that is converted to BC (i.e. bacterioplankton growth efficiency). Thus, understanding how *p*CO_2_ affects the carbon content of bacterioplankton cells enabled us to extend our results beyond carbon loss via respiration, to the partitioning of surface organic carbon between dissolved (that which escapes or resists bacterioplankton consumption) and particulate (as BC) organic carbon.

**Fig 7:**
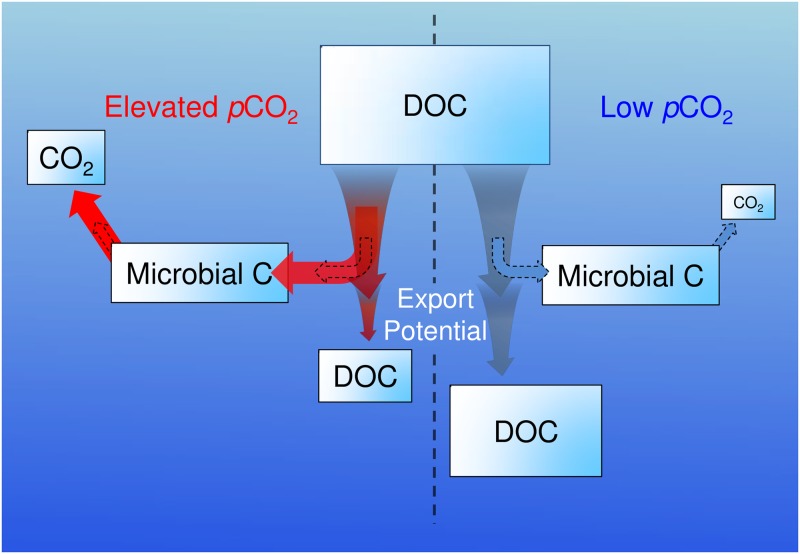
Effects of *p*CO_2_ on bacterioplankton removal of organic carbon. A depiction of the short-term effects of *p*CO_2_ on the use of DOC by bacterioplankton communities in ocean surface waters. Elevated *p*CO_2_ may increase the use of DOC at lowered bacterioplankton growth efficiencies by natural bacterioplankton communities, increasing the respiration of DOC and decreasing the magnitude of accumulated DOC in these regions, ultimately decreasing the amount of DOC available for vertical export (i.e. export potential). Arrows represent the flux of carbon between identified pools. Red represents processes occurring under elevated (> ambient) *p*CO_2_ conditions while blue represents processes occurring under low (ambient or pre-industrial) *p*CO_2_ conditions. Dotted black arrows denote the flux of carbon between identified pools at low *p*CO_2_ to enable an easy comparison between elevated and low *p*CO_2_ fluxes.

We observed no direct effect of *p*CO_2_ on the carbon content of bacterioplankton cells. Assuming this equivalency across all experiments, we calculated bacterioplankton carbon demand for all of the experiments where a change in TOC was resolvable. Bacterioplankton carbon demand was estimated as the change in DOC from the start of the incubations to stationary growth phase and DOC was estimated as TOC—BC. These calculations revealed a consistent pattern across DOC amendments and experimental location: bacterioplankton communities grown at elevated *p*CO_2_ exhibited enhanced carbon demand relative to communities at pre-industrial and ambient *p*CO_2_ (Fig 5). These results suggest that bacterioplankton communities exposed to short-term increases in *p*CO_2_ reduce the amount of DOC available for vertical export through enhanced consumption of recently produced DOC (Fig 7).

Estimates of bacterioplankton growth efficiency revealed a consistent pattern across DOC amendment and experimental location: growth efficiencies were consistently lower at elevated *p*CO_2_ than at pre-industrial or ambient *p*CO_2_ (Fig 6). This implies that a greater portion of consumed DOC is converted to CO_2_ by bacterioplankton communities exposed to elevated *p*CO_2_ (Fig 7). It is important to note that variable initial bacterioplankton communities likely resulted in variable BC and that our estimate of cellular carbon conversion factor may not accurately represent communities across experiments; however, only the absolute magnitude of the growth efficiency values will be affected if, as our results suggest, BC is similar across *p*CO_2_ levels. Enhanced bacterioplankton respiration at elevated *p*CO_2_ will consistently result in lower growth efficiencies, regardless of the exact carbon conversion factor used.

Our estimates of bacterioplankton carbon demand and growth efficiency demonstrate a direct effect of *p*CO_2_ on bacterioplankton processing of organic carbon in the surface ocean. While elevated *p*CO_2_ had little impact on BC production, enhanced DOC removal through accelerated bacterioplankton consumption and reduced growth efficiencies reduced the amount of DOC available for export, relative to pre-industrial *p*CO_2_ conditions (Fig 7). Enhanced removal of DOC could also lead to increased production of recalcitrant organic compounds via the microbial carbon pump [54]. Further experiments and measurements of long-term DOC removal and DOC characterization are required to evaluate this possibility.

### Possible mechanisms

Consumption of organic carbon is often a function of bacterioplankton community structure and a number of studies have demonstrated that slight variations in DOC may select for specific bacterioplankton populations over timescales of hours to days [28, 40, 53, 55]. It is likely that bacterioplankton community composition shifted over time in our experiments and it is also possible that a shift in community composition between *p*CO_2_ treatments contributed to the consistent differences in TOC removal. A recent study by Siu et al. [17] indicated that elevated *p*CO_2_ (~1050 ppm) could induce shifts in bacterioplankton composition. However, *p*CO_2_ manipulations in mesocosm experiments in Bergen [56, 57] and Svalbard, Norway [58–60] showed that the free-living bacterioplankton community structure was mostly unaffected by elevated *p*CO_2,_ which ranged from ~750–1085 ppm, depending on the experiment. In the present study the trend of enhanced bacterioplankton respiration in the presence of elevated *p*CO_2_ was observed across DOC experiments for which water was collected at various times of the year and presumably contained different initial bacterioplankton communities. While it is possible that the bacterioplankton community structure shifted under elevated *p*CO_2_ conditions, the response of enhanced respiration at elevated *p*CO_2_ appeared universal despite likely differences in initial microbial composition sampled across sites and time.

Alternatively, enhanced bacterioplankton respiration combined with low bacterioplankton growth efficiency in elevated *p*CO_2_ treatments may be a physiological response to decreased pH. A recent study examined the expression of bacterioplankton transcripts in response to elevated *p*CO_2_ and showed that transcripts associated with respiration were significantly enhanced in elevated *p*CO_2_ mesocosms [61]. The authors specifically showed upregulation of respiratory proton pumps that aid in translocating protons across the cell membrane. These results suggest that as environmental pH decreases, heterotrophic bacterioplankton upregulate respiratory proton pumps to export protons that invade the cell as a result of low external pH. Consistent with our observations, Bunse et al. [61] suggested that upregulating proton pumps under low pH would be energetically costly to heterotrophic bacterioplankton and thus reduce their overall efficiency.

There is also evidence to suggest that increasing *p*CO_2_ may lead to increased removal of organic matter in the ocean through up-regulation and/or enhanced efficiencies of extracellular enzymes [9–12]. Extracellular enzymes convert high molecular weight organic compounds to low molecular weight compounds that can be used by heterotrophic bacterioplankton [62–64]. These enzymes are not buffered by the cell’s cytoplasm and are directly impacted by external changes in pH. An increase in the concentration of hydrogen ions in an enzyme’s environment, as a result of declining pH, may alter the three-dimensional protein structure of the enzyme’s active site and thus affect enzymatic activity [65, 66]. Recent studies report that extracellular α and β-glucosidase activity increased in response to elevated hydrogen ion concentration, suggesting that a decline in pH of 0.2–0.3 pH units was closer to the pH optimum of glucosidase activity than ambient pH [11, 12]. It may be that up-regulation of enzymes like α and β-glucosidase by marine heterotrophic bacterioplankton in conjunction with altered enzymatic active sites led to the increased removal of DOC measured within elevated *p*CO_2_ treatments. Further experimentation is required to explore these mechanisms.

Thus, while we do not exclude the possibility of a shift in the bacterioplankton community under elevated *p*CO_2_ conditions, we suggest that the increased respiration at elevated *p*CO_2_ is likely due to up-regulation of proton pumps and possibly the enhancement of extracellular enzyme activity (via up-regulation and/or enhanced efficiencies).

### Conclusions

This study reveals a direct impact of *p*CO_2_ on bacterioplankton removal of organic carbon. In all experiments for which TOC removal was measurable, enhanced bacterioplankton respiration under elevated *p*CO_2_ coupled with minimal change in bacterioplankton biomass yield resulted in systematically greater cell-specific respiration in elevated *p*CO_2_ treatments (i.e. cell-specific TOC removal; Fig 4). Even in cases where bacterioplankton abundance yield was greater under elevated *p*CO_2_, enhanced respiration led to greater amounts of carbon respired per cell, increasing the likelihood that surface organic carbon will be lost to the atmosphere through outgassing of CO_2_. Estimates of bacterioplankton carbon demand and growth efficiency suggest that acidified conditions may also increase the ability of bacterioplankton to consume DOC but with reduced growth efficiencies. The cumulative effect of enhanced consumption and enhanced respiration under elevated *p*CO_2_ would increase the fraction of surface-ocean DOC respired to CO_2_, ultimately decreasing the effectiveness of DOC as a sink of carbon in the ocean (Fig 7). Incorporation of these results into numerical models will enable more accurate understanding of current air-sea carbon exchange and the impact of elevated *p*CO_2_ in surface waters on biogeochemical cycles.
